# Quartz Microcrystal-Hybridized Organosilicone Encapsulant with Enhanced Optical and Thermal Performances

**DOI:** 10.3390/polym10010084

**Published:** 2018-01-16

**Authors:** Xin Chen, Yancong Feng, Xiao Wang, En Li, Yao Wang, Lingling Shui, Hao Li, Nan Li, Guofu Zhou

**Affiliations:** 1 Guangdong Provincial Key Laboratory of Optical Information Materials and Technology & Institute of Electronic Paper Displays, South China Academy of Advanced Optoelectronics, South China Normal University, Guangzhou 510006, China; chenxinchauncey@163.com (X.C.); fengyancong@m.scnu.edu.cn (Y.F.); wang2009xiao@163.com (X.W.); lien1016@163.com (E.L.); wangyao@m.scnu.edu.cn (Y.W.); shuill@m.scnu.edu.cn (L.S.); 2 National Center for International Research on Green Optoelectronics, South China Normal University, Guangzhou 510006, China; 3 Shenzhen Guohua Optoelectronics Tech. Co. Ltd., Shenzhen 518110, China; nan.li@guohua-oet.com; 4 Academy of Shenzhen Guohua Optoelectronics, Shenzhen 518110, China

**Keywords:** organosilane, quartz microcrystal, encapsulant, refractive index, thermal conductivity

## Abstract

Encapsulant is one determining factor underpinning the device lifetimes of organic optoelectronics. However, encapsulant seriously needs improvement in optical, thermal, and mechanical properties, especially to develop organic light emitting diodes. In this study, we prepared an in situ crosslinked organosilane composite containing benzyloxy and glycidyl-modified quartz microcrystal (mQMC) as high performance encapsulant. In the present work, methylphenylsilanediol (MPSD) was introduced as a novel crosslinker to impart appropriate structural strength. Along with increasing mQMC fillers, this organosilane system shows improved properties, such as refractive index, thermal stability, and storage modulus. Specifically, these hybridized mQMCs in the organosilane framework may facilitate an approximate two-fold increase (0.238 W/(m·K)) in overall thermal conductivity at the determined concentration.

## 1. Introduction

In recent years, organic electronics have attracted more and more interest in a range of fields, e.g., display, sensor array, lighting panel, photovoltaic module and so on. As the first commercial application, organic light emitting diodes (OLEDs) reduce energy consumption and decrease device complexity compared to conventional liquid crystal displays [1]. However, their extremely sensitive components remarkably weaken device reliability. Naturally, encapsulant becomes one determining factor to provide hermetic protection for OLEDs. In this process, optical, thermal, mechanical, water, and oxygen barriers are involved in the specific demands of encapsulant performance [2]. 

As a frame material, epoxy is widely used as encapsulant due to its good adhesion, strong mechanical strength and rapid curing processing, but is still limited by poor moisture resistance, weak thermal stability, and a low refractive index [3,4,5,6,7]. On the contrary, organosilicone resin possesses a relatively high refractive index, strong moisture resistance, and good thermal stability, as well as good compatibility with glass substrate [8,9,10,11,12]. Therefore, organosilicone has gradually become a more suitable encapsulant candidate with high performance. 

However, the modification or formula regulation of frame material falls short of the demands for an excellent OLED encapsulant. Typically, the introduction of high polarization groups or atoms to the matrix [13,14,15,16]—such as phenyl group [8,17,18,19,20,21,22], sulfur [15], fluorine [12,17], phosphor [18], and nitrogen [18]—can effectively heighten the refractive index. On the other hand, some nanoparticles—e.g., TiO_2_ [19,23] and ZrO_2_ [3]—can be also introduced to achieve a desirable refractive index of encapsulant by hybridization. At the same time, the addition of similar nanoparticles—such as boron nitride nanosheets [7,24,25], graphene nanosheets [9,10], black phosphorus [26], ZnO [27], silicon carbide [28], core-shell Ag@SiO_2_ [29], and carbon nanotube [30]—will contribute significantly to the thermal conductivity of encapsulant materials.

In this work, we chose cheap and compatible quartz microcrystal (QMC) as filler and crosslinkers, and chose novel methylphenylsilanediol (MPSD) as molecular crosslinker, to construct a highly in situ crosslinked organosilicone network. Hereinto, surface modification of QMC will not only bring abundant phenyl groups to this organosilicone system for high refractive index, but also increase crosslinking density for good mechanical strength and thermal stability. Especially, high thermal conductivity of QMC [8 W/(m·K)] also facilitates overall thermal diffusion in the system. Moreover, MPSD will favor appropriate structural rigidity of organosilicone encapsulant.

## 2. Materials and Methods 

### 2.1. Materials

Dichloromethylphenylsilane (98%), 3-glycidyloxypropyl trimethoxysilane (97%, GMS), vinyltrimethoxysilane (>98%, VTMS), and methyldiethoxysilane (>98%, MDES) were all provided by J&K Chemical Technology (Beijing, China). Barium hydroxide monohydrate (97%, Ba(OH)_2_·H_2_O) was purchased from HWRK Chemical (Beijing, China). Quartz microcrystal (mean size: ~5 μm) was bought from Lianyungang Donghai Silica Powder Co., Ltd. (Jiangsu, China). Both Pt Karstedt’s catalyst (Platinum(0)-1,3-divinyl-1,1,3,3-tetramethyldisiloxane complex solution in *p*-xylene (~2% Pt), Sigma-Aldrich, St. Louis, MO, USA) and Amberlite IRC76 (Alfa Aesar, Haverhill, MA, USA) were directly used. All other reagents and organic solvents are of analytic reagent (AR) without further treatment prior to use.

### 2.2. Synthesis of MPSD

**Figure 1:**
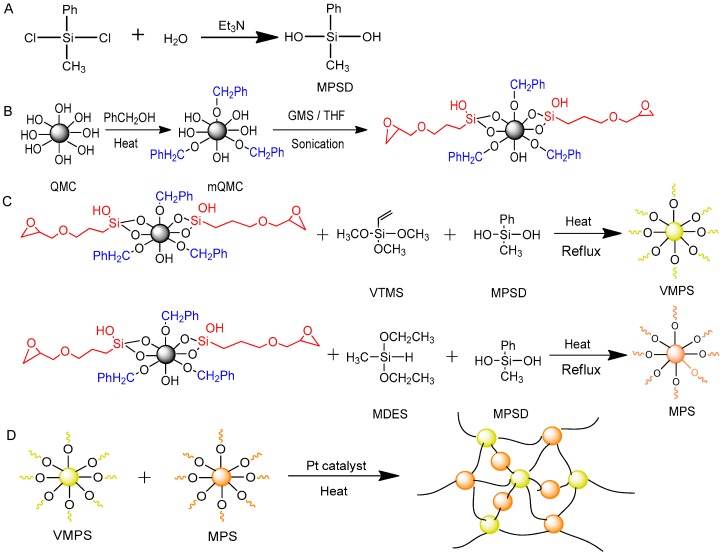
Synthesis routes of (**A**) MPSD; (**B**) surface-modified QMC; (**C**) two intermediate resins—VMPS and MPS; and (**D**) final quartz–organosilane composite.

MPSD was obtained by hydrolysis reaction (see Figure 1A) [31]. Typically, dichloromethylphenylsilane was dropwise added to the mixture of triethylamine, acetone, ultrapure water, and ether for 1 h while stirring at 0 °C. Then, the redundant triethylamine hydrochloride was removed by vacuum filtration. Subsequently, the resulting filtrate was concentrated to one-tenth of the initial volume and then precipitated in excess cold *n*-hexane. After freezing for 24 h, the final MPSD was separated out and collected by vacuum filtration. 

### 2.3. Modification of Quartz Microcrystal 

Both a determined amount of QMC and excess benzyl alcohol were added into a stainless vessel and then heated up to 240 °C for 96 h. Afterwards, benzyloxy-modified QMCs were obtained by high speed centrifugation for 10 min. Next, these QMCs were mixed fully with GMS in tetrahydrofuran under sonication for 2 h. Finally, all the products, benzyloxy, and glycidyl-modified QMC (mQMC) were concentrated to 5 mL in dry argon flow (see Figure 1B) [3].

### 2.4. Synthesis of Vinylmethylphenylsiloxane Resin (VMPS) 

VMPS was obtained by sol-gel condensation of VTMS and MPSD (molar ratio: 1:1) in *p*-xylene (10 wt. %) at 80 °C while stirring for 4 h, using Ba(OH)_2_·H_2_O (0.1 mol %) as catalyst (see Figure 1C) [32]. After the reaction proceeded for 2 h, the catalyst was filtered out with a 10 μm syringe filter. The dropwise addition of mQMC into the reacting mixture followed immediately. Finally, all the volatile components were removed in vacuum.

### 2.5. Synthesis of Methylphenylsiloxane Resin (MPS)

Similarly, MPS was synthesized by sol-gel condensation of MDES and MPSD (molar ratio: 1:1.5) at 100 °C while stirring for 12 h, using Amberlite IRC76 (0.1 mol %) as acidic catalyst (see Figure 1C) [32]. Once the reaction proceeded for 2 h, the catalyst was filtered out with a 10 μm syringe filter. Then the dropwise addition of mQMC into the reacting mixture followed immediately. Finally, all the volatile components were removed in vacuum. 

### 2.6. Fabrication of Quartz−Organosilicone Composite 

The quartz−organosilicone composite was synthesized by hydrosilylation of VMPS and MPS using Pt Karstedt’s catalyst while stirring for 2 h (see Figure 1D) [32]. Afterwards, the mixture was homogenized by sonication for 1 h and then cast into a customized stainless steel mold. Finally, the sample was cured at 90 °C for 1 h, followed by heating at 150 °C for 2 h in air. 

### 2.7. Chemical Characterizations

The chemical structure of MPSD was tested by electron ionization mass spectrometer (EI-MS) using a NexION 300 supplied by PerkinElmer (Waltham, MA, USA). The surface modification of QMC was identified by Fourier transform infrared spectrometer (FTIR) using a Vertex 70 supplied by Bruker (Karlsruhe, Germany) in a spectral range of 4000–400 cm^−1^ with the resolution of 2 cm^−1^. Samples were prepared using KBr plate method.

### 2.8. Optical Measurements 

The transmittances of the final quartz−organosilicone composite films were measured by ultraviolet–visible–near infrared spectrophotometer (UV–vis–NIR) using a LAMBDA 950 supplied by PerkinElmer in the wavelength range of 400–700 nm. All the thicknesses of the samples were 1 mm. The measurements of refractive index were performed by refractometer using a DR-2 supplied by ATAGO (Tokyo, Japan) at 589 nm and 25 °C.

### 2.9. Thermal Analysis

The thermal stability of the samples was examined by thermal gravimetric analysis (TGA) using a TGA 2 supplied by METTLER TOLEDO (Greifensee, Switzerland) in the temperature range from 25 °C to 1000 °C with a heating rate of 10 °C/min in nitrogen atmosphere (flow rate: 50 mL/min). 

The thermal conductivity was determined by laser thermal conductivity analysis using a LFA447 supplied by NETZSCH (Selb, Germany) at 25 °C. All the sizes of samples were 10 × 10 mm^2^ with the thickness of 1 mm.

### 2.10. Mechanical Test

The mechanical performance was tested by dynamic mechanical thermal analysis (DMA) using a DMA 1 supplied by METTLER TOLEDO (Greifensee, Switzerland) in a temperature range from 50 °C to 260 °C with a heating rate of 5 °C/min at 1 Hz in the shear mode. 

## 3. Results and Discussion

**Figure 2:**
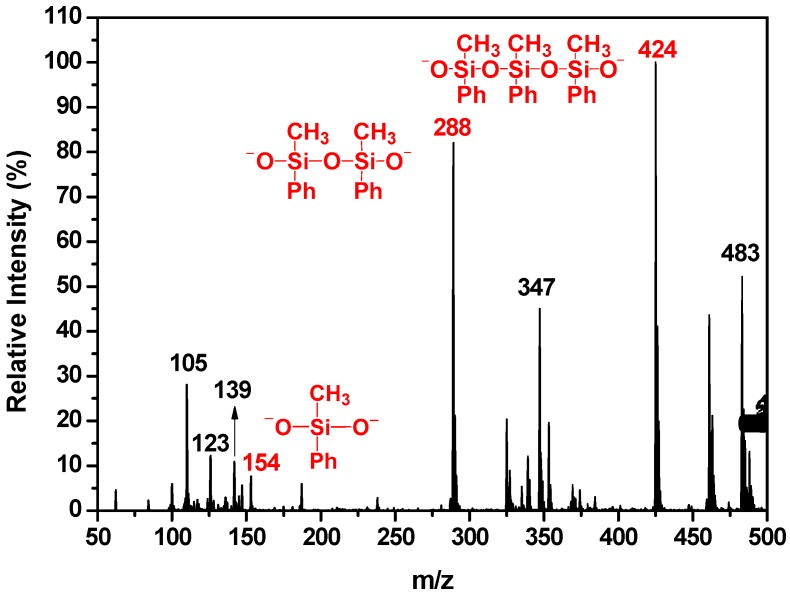
Mass spectrum of MPSD. The related peaks list below: 105 (*m*/*z*, SiPh), 123 (*m*/*z*, SiOPh), 139 (*m*/*z*, Si(OH)_2_Ph), 154 (*m*/*z*, SiO_2_PhCH_3_), 288 (*m*/*z*, Si_2_O_3_Ph_2_C_2_H_6_), 347 (*m*/*z*, Si_3_O_4_Ph_2_C_3_H_9_), 424 (*m*/*z*, Si_3_O_4_Ph_3_C_3_H_9_), 483 (*m*/*z*, Si_4_O_5_Ph_3_C_4_H_12_).

As the best candidate for encapsulant, organosilicone still needs to be improved in optical and thermal performances. In the present work, both MPSD crosslinker and QMC additive were introduced into the siloxane matrix by four-step fabrication (see Figure 1). The related descriptions of fabrication are presented in the Materials and Methods section and Supporting Information in detail. MPSD was obtained by hydrolysis reaction as shown in Figure 1A. Hereinto MPSD is so active that reacts to form a series of different oligomers. As shown in Figure 2, there are a dimer peak at 288 *m*/*z*, and a trimer peak at 424 *m*/*z*, much stronger than the monomer peak at 154 *m*/*z*. Although the obtained product is a mixture of MPSD and its oligomers, their good solubility in two silane monomers—VTMS and MDES—contribute greatly to the following coupling reactions.

**Figure 3:**
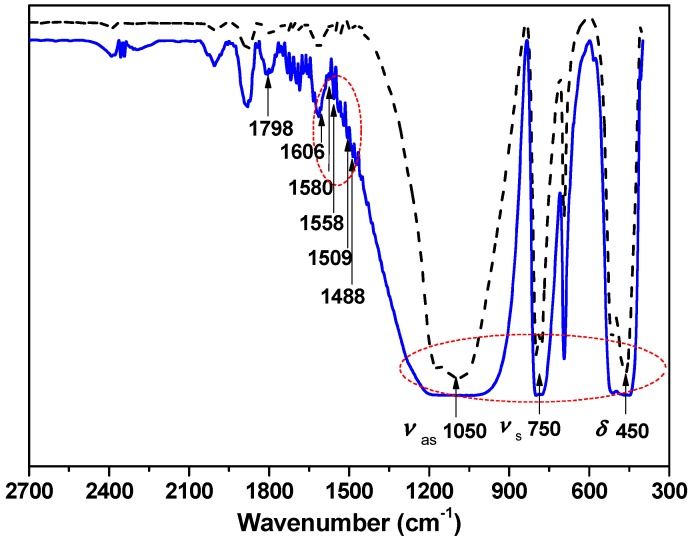
FTIR spectra of quartzes before and after surface modification with benzyl alcohol. The dash black line is quartz without modification and the solid blue line is the modified quartz. The related peaks list below: 450 cm^−1^ (δ, –Si–O linked with quartz microcrystal), 750 cm^−1^ (ν_s_, –Si–O linked with quartz microcrystal), 1050 cm^−1^ (ν_as_, –Si–O linked with quartz microcrystal), 1488 cm^−1^, 1509 cm^−1^, 1558 cm^−1^, 1580 cm^−1^, 1606 cm^−1^ (–C_6_H_5_ linked with benzyl alcohol), 1798 cm^−1^ (solvent THF).

In fact, benzyloxy- and glycidyl-modified QMC (mQMC) (see Figure 1B) also act as another kind of crosslinker with extremely high crosslinking intensity. Figure 3 obviously displays the absorption bands of benzyl groups at 1400–1600 cm^−1^ [3]. This proves that the benzyl group is successfully grafted to the surface of QMC. It may effectively enhance the compatibility of QMC with the organosilicone frameworks, owing to similar chemical groups.

**Figure 4:**
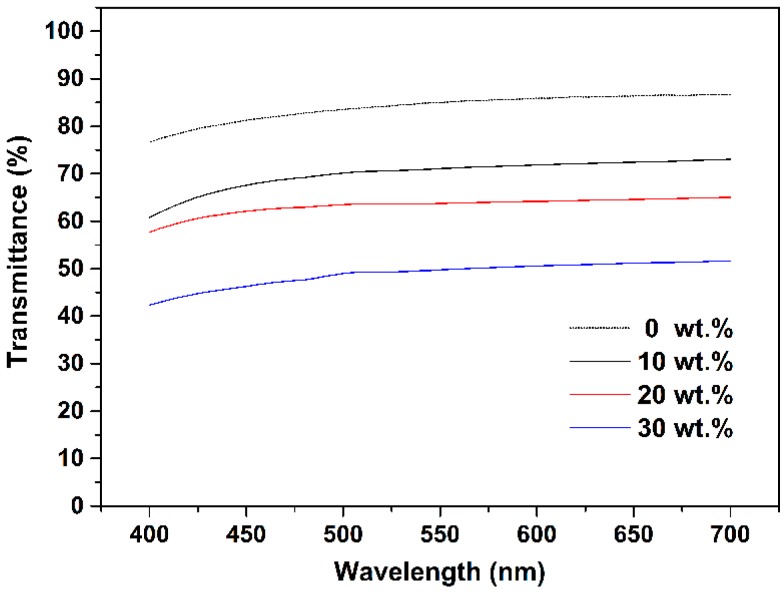
Transmittance curves of quartz–organosilicone composites with different mQMC contents in the visible spectrum.

At the same time, abundant glycidyl groups on the surface of mQMC—joined together with Si–H functional bonds, vinyl groups, and MPSD—constructed a highly crosslinked network. Naturally, this predominant organosilicone nature will assign good optical properties to the quartz composites. As shown in Figure 4, transmittance of the neat organosilicone reaches nearly 90% in the visible range from 450 nm to 700 nm. However, the high refractive index of mQMC is adverse to transparent blank matrix. Thus, along with increasing mQMC content, the composite exhibits a decreasing transmittance in the range of visible light.

**Figure 5:**
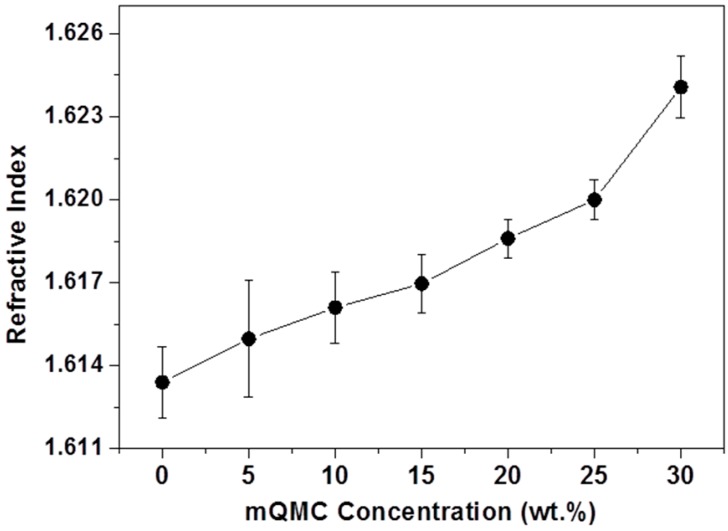
Refractive indexes of quartz−organosilicone composites with different mQMC contents.

Contrary to the falling transmittance, the refractive index of quartz–organosilicone composite rises from initial 1.613 to 1.623 at the loading of 30 wt. %. Surprisingly, the refractive index of the neat sample reaches a very high level of over 1.6, which is much larger than that of siloxane-hybridized materials with high refractive index in the previously reported works [8,20]. This performance may mainly attribute to the crosslinker we selected. According to the classic electromagnetic theory, the refractive index is derived from Lorentz–Lorenz equation [33]

(1) $$P=\frac{n^{2}-1}{n^{2}+1}\times \frac{M}{\rho }$$

where *P* is the molar polarization, *n* is the refractive index of quartz−organosilicone, *M* is the molecular weight, and *ρ* is the density. Obviously, the refractive index has a positive correlation with the material polarization. As the key crosslinker, MPSD gives this framework plenty of benzyl groups to improve material polarization, which explains the extremely high refractive index of blank sample. On the other hand, as the content of filler increases, more and more benzyl groups of mQMC are introduced into the composite. Of course, high refractive index of mQMCs themselves is also a favorable factor. As revealed in Figure 5, the refractive index shows a good liner relation with the content of mQMC. It agrees well with the idea that the chemical structure is a critical factor in enhancing the refractive index of materials from the formula above [33]. 

**Figure 6:**
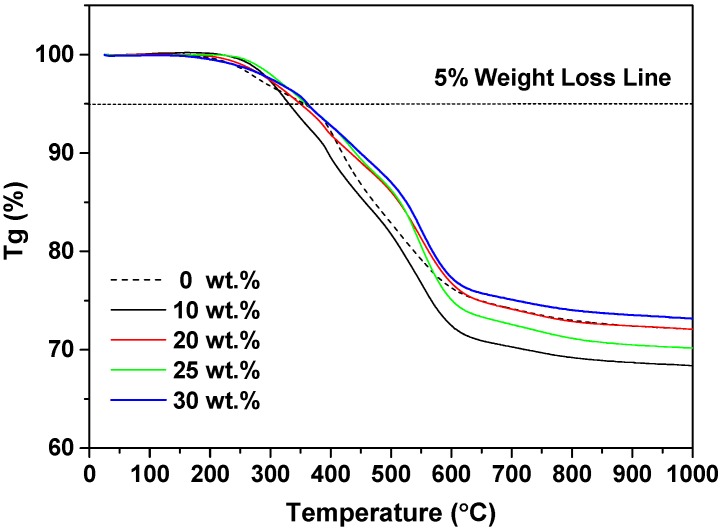
Thermogravimetric curves of quartz−organosilicone composites with different mQMC contents.

Afterwards, the thermal performances of this highly crosslinked organosilicone network, including thermal stability and conductivity, are evaluated in detail. Figure 6 firstly exhibits high thermal stability of quartz−organosilicone composites. The derivatives of thermal spectra are presented in Figure S1. In Figure 6, for blank sample, 5% weight loss temperature is 356 °C, much higher than the common encapsulants [6]. As the content of mQMC filler rises above 20 wt. %, a 5% weight loss temperature develops from 356 °C (0 wt. %) to 361 °C (25 wt. % and 30 wt. %). This is different from the ZrO_2_ particle which is able to disturb the crosslinking network [3].

**Figure 7:**
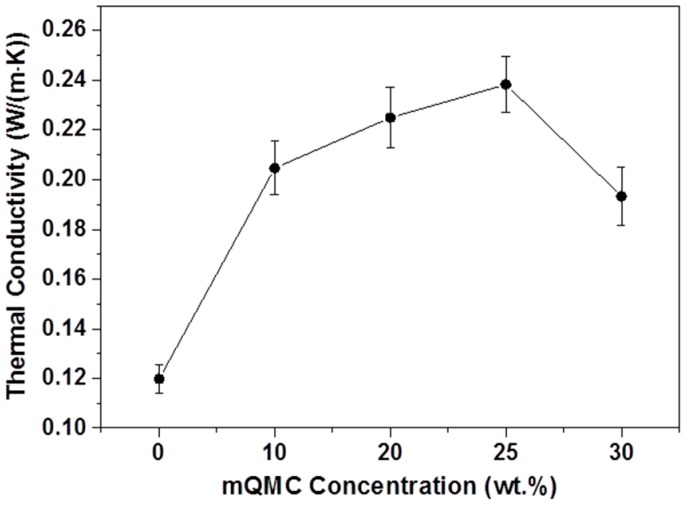
Thermal conductivities of quartz−organosilicone composites with different mQMC contents.

Except thermal stability, heat diffusion is a serious problem for encapsulant which influences the performance of electronic devices even the lifetime. For improving thermal conductivity of encapsulant, QMC (about 8 W/(m·K)) is specifically chosen as the critical filler in this composite. Figure 7 illustrates that, the thermal conductivity of quartz−organosilicone composite reaches to the peak at the mQMC content of 25 wt. %. It may be ascribed to the distribution of highly thermoconductive QMC in the organosilicone network. At the low concentration, mQMCs are well-distributed in the matrix to make the heat flux homogeneous. Here, the enhancement of thermal conductivity is obvious. However, once the mass ratio of mQMC filler achieves 30 wt. %, the aggregation of particles emerges, leading to a decrease of thermal conductivity.

**Figure 8:**
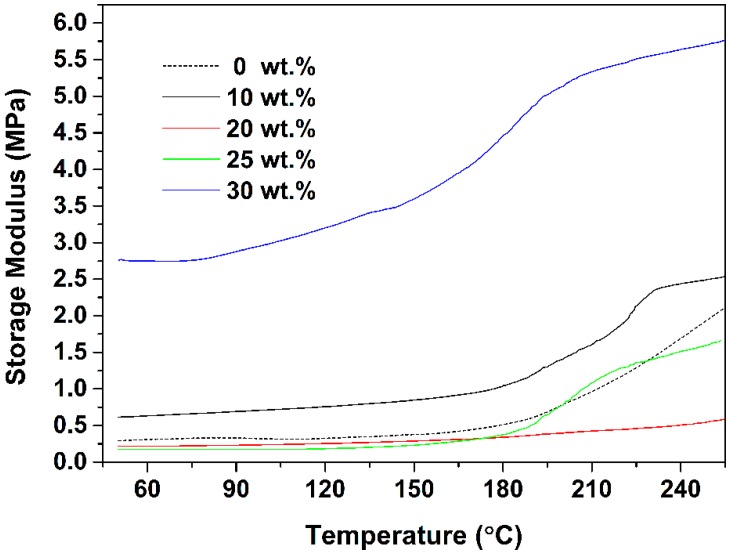
Dynamic mechanical thermal analyses of quartz−organosilicone composites with different mQMC contents in shear mode.

The last key parameter, the mechanical performances of quartz−organosilicone encapsulants, are represented in Figure 8. Remarkably, the highest storage modulus is obtained at the mQMC content of 30 wt. %. As the mQMC content below 30 wt. %, the addition of fillers does not bring any obvious variation of storage modulus. Once the content of filler reaches 30 wt. %, the crosslinking network has formed and thus a sharp increment of storage modulus emerges, presenting an obvious reinforcing effect. Meanwhile, the TGA curves in Figure 6 also indicate that, the 30 wt. % loading is enough to form a dense crosslinking network. Furthermore, the high storage modulus reveals that this kind of crosslinking quartz−organosilicone composite is strong enough to resist outer pressure during encapsulation.

## 4. Conclusions

In this study, both compatible QMC and novel crosslinker MPSD were introduced into one in situ crosslinked organosilane matrix as high performance encapsulant. In this case, surface modified QMC as high density crosslinks can greatly increase the crosslinking degree of the organosilane composite to improve thermal stability and mechanical performance. Their high thermal conductivity also facilitates the overall thermal diffusion in the system with about double the coefficient of heat conductivity (0.238 W/(m·K)). The only drawback is the falling transmittance along with increasing mQMC feeds. Moreover, MPSD possesses a more appropriate molecular rigidity compared with common diphenyl silanediol and dimethyl silanediol, so that it can integrate numerous phenyls on the surface of QMC into the organosilane encapsulant to create a very high refractive index (~1.623) and greatly enhance storage modulus. In view of cost, performance, and system compatibility, this highly crosslinked quartz−organosilane composite is anticipated to be a good encapsulant candidate for improving reliability of OLEDs. 

## Supplementary Materials

The following are available online at www.mdpi.com/2073-4360/10/1/84/s1, Figure S1: Thermogravimetric curves of quartz-organosilicone composites with different mQMC contents. (**a**) 0 wt.%, (**b**) 10 wt.%, (**c**) 20 wt.%, (**d**) 25 wt.% and (**e**) 30 wt.%.
