# Supplementary material for: Quartz Microcrystal-Hybridized Organosilicone Encapsulant with Enhanced Optical and Thermal Performances
Source: Polymers (Basel). 2018 Jan 16;10(1):84. doi: 10.3390/polym10010084 (PMC6414841; doi:10.3390/polym10010084)
Supplement: Supplementary file 1 [file polymers-10-00084-s001.pdf]

Supporting Information for "Quartz microcrystal-hybridized Organosilicone Encapsulant with Enhanced Optical and Thermal Performances"

Xin Chen[1,2,†], Yancong Feng[1,2,†], Xiao Wang[1,2], En Li[1,2], Yao Wang[1,2], Lingling Shui[1,2], Hao Li[1,2,*], Nan Li[3,4] and Guofu Zhou[1,2,3,4,*]

[1] Guangdong Provincial Key Laboratory of Optical Information Materials and Technology & Institute of Electronic Paper Displays, South China Academy of Advanced Optoelectronics, South China Normal University, Guangzhou 510006, P. R. China; chenxinchauncey@163.com (X.C.); fengyancong@m.scnu.edu.cn (Y.F.); wang2009xiao@163.com (X.W.); lien1016@163.com (E.L.); wangyao@m.scnu.edu.cn (Y.W.); shuill@m.scnu.edu.cn (L.S)

[2] National Center for International Research on Green Optoelectronics, South China Normal University, Guangzhou 510006, P. R. China

[3] Shenzhen Guohua Optoelectronics Tech. Co. Ltd., Shenzhen 518110, P. R. China; nan.li@guohua-oet.com

[4] Academy of Shenzhen Guohua Optoelectronics, Shenzhen 518110, P. R. China

* Correspondence: haoli@scnu.edu.cn (H.L.); guofu.zhou@m.scnu.edu.cn (G.Z.); Tel.: +86-20-39314813.

† These authors contributed equally to this work.

Synthesis of quartz-organosilane composite

Figure 1 in the manuscript shows the four-step fabrication of quartz-organosilane composite. Figure 1A shows the fabrication of the cross-linker, methylphenylsilanediol (MPSD). It is mainly about a hydrolysis reaction of dichloromethylphenylsilane. Figure 1B is the surface modification of quartz microcrystal, mixing the quartz microcrystal with benzyl alcohol while heating. After heating, the couple agent is added into the mixture with THF and sonication. Figure 1C is the in-situ reaction of vinyltrimethoxysilane (VTMS), cross-linker methylphenylsilanediol (MPSD) and modified quartz microcrystal. Another is the in-situ reaction of methyldiethoxysilane (MDES), cross-linker methylphenylsilanediol (MPSD) and modified quartz microcrystal. Figure 1D is the hydrosilylation of two products derived from Figure 1C.

Thermal Analysis

The thermal stability of the samples was examined by thermal gravimetric analysis (TGA) using a TGA 2 supplied by METTLER TOLEDO (Switzerland) in the temperature range from 25 °C to 1000 °C with a heating rate of 10 °C/min in nitrogen atmosphere (flow rate: 50 mL/min). Figure S1 shows the thermogravimetric curves of quartz-organosilicone composites with different mQMC contents.

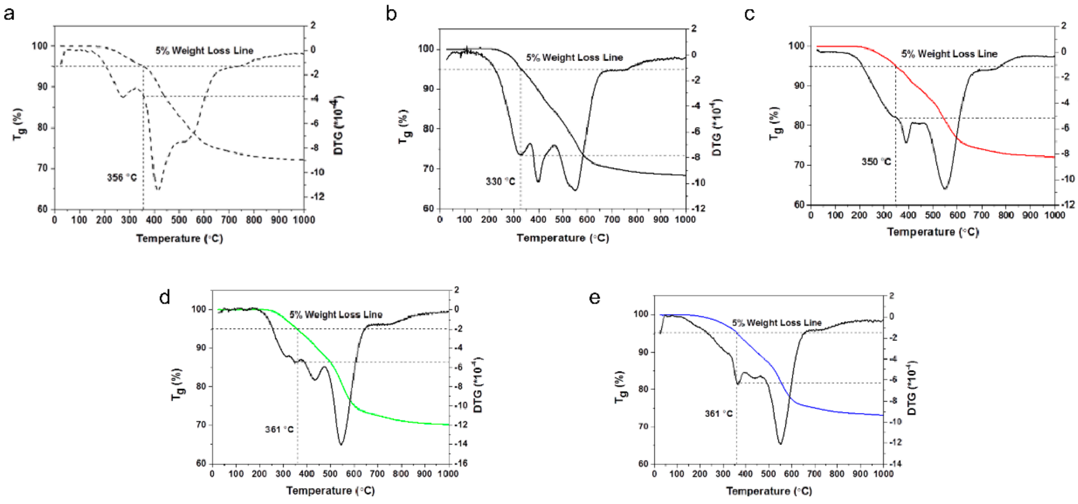

Figure S1. Thermogravimetric curves of quartz-organosilicone composites with different mQMC contents. (**a**) 0 wt.%, (**b**) 10 wt.%, (**c**) 20 wt.%, (**d**) 25 wt.% and (**e**) 30 wt.%.
